# Hypercalcemia in Pregnancy Caused by a Uterine Myoma

**DOI:** 10.1210/jcemcr/luae126

**Published:** 2024-08-14

**Authors:** Stephanie van der Leij, Doenja Hertog

**Affiliations:** Department of Endocrinology, University Medical Center, Utrecht University, Utrecht, CX 3584, The Netherlands; Princess Máxima Center for Pediatric Oncology, Utrecht, CS 3584, The Netherlands; Department of Endocrinology, University Medical Center, Utrecht University, Utrecht, CX 3584, The Netherlands

**Keywords:** parathyroid hormone-related peptide, hypercalcemia, pregnancy, uterine myoma

## Abstract

We present a case of a PTH-related peptide (PTH-rp) producing uterine myoma, leading to hypercalcemia in pregnancy. Our patient presented with dehydration, hypotension, delirium, and malnutrition. Due to a serum calcium level of 17.9 mg/dL (4.48 mmol/L) (reference range 8.8-11.2 mg/dL; 2.20-2.80 mmol/L), prompt treatment with hydration and calcitonin was initiated. The patient went into labor before we could consider other treatment options. Although uncommon in pregnancy, it is of great importance to identify hypercalcemia since it is related to a high risk of maternal and neonatal morbidity and mortality. Because bisphosphonates are contraindicated in pregnancy, hydration and calcitonin are the cornerstones of treatment for PTH-rp-induced hypercalcemia.

## Introduction

Hypercalcemia in pregnancy is rare, and primary hyperparathyroidism, which affects about 0.03% of women of reproductive age, tends to be the primary cause. Prompt diagnosis and treatment of hypercalcemia in pregnancy is of great importance because of the high risk of maternal and neonatal morbidity and mortality (1-3). It can be challenging to diagnose hypercalcemia in pregnancy because symptoms of hypercalcemia can overlap with symptoms of pregnancy. It also requires knowledge of calcium physiology, which changes during pregnancy. During pregnancy, the total calcium level declines due to volume expansion, while ionized calcium levels remain within the normal range. Up to 30 grams of calcium is transported from the mother to the fetus to enable fetal bone development. Active vitamin D, which is upregulated more than 2-fold during pregnancy, stimulates intestinal calcium absorption in order to fulfill the increased calcium demand. Maternal PTH is low to normal in pregnancy, but PTH-related peptide (PTH-rp) increases, primarily from its production by the placenta and the breast. PTH-rp is responsible for the active placental calcium transport to the fetus, particularly in the third trimester (3, 4). Interestingly, PTH-rp is also expressed in myoma tissue (5). We present a case of a pregnant woman with hypercalcemia caused by the production of PTH-rp by a uterine myoma.

## Case Presentation

A 45-year-old woman, 31 weeks pregnant, was admitted to our emergency room due to dehydration with hypotension and tachycardia, delirium, and malnutrition. She had been suffering from increased physical weakness, anorexia, and hallucinations for several weeks.

**Figure 1.**
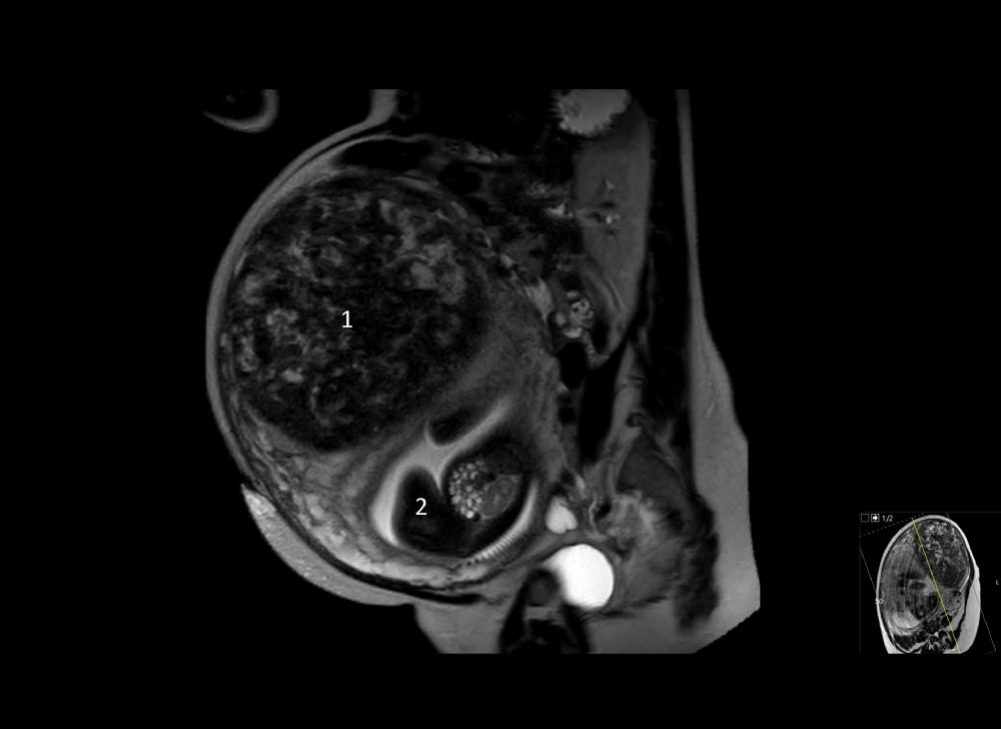
Sagittal magnetic resonance imaging at 31 weeks of pregnancy with uterus myoma (1) 7.9 × 8.7 inches (20 × 22 cm) and fetus dorsocaudal in uterus (2).

Her medical history revealed a uterine myoma 7 years earlier, which in 2 years had grown from 8 to 15 cm (3.2 to 5.9 inches), for which a hysterectomy had been advised to rule out leiomyosarcoma. The patient refused treatment, however, and was lost to follow-up. She used no medication except for daily vitamin D 1600 international units. Her clinical symptoms were explained by her blood results, which showed PTH-rp-mediated hypercalcemia (Table 1). An abdominal ultrasound showed a normal-sized fetus according to gestational age and no signs of placenta abruption; a computed tomography scan showed no signs of a pulmonary embolism or a tumor, and abdominal magnetic resonance imaging showed no changes of the uterine myoma (Fig. 1).

**Table 1.** Laboratory results at presentation

	Value, SI units	Value, conventional units	Reference ranges (SI units and conventional units)
Hemoglobin	93.5 g/L	9.35 g/dL	119.2-154.7 g/L (11.9-15.5 g/dL)
Creatinine	80 µmol/L	0.90 mg/dL	45-90 µmol/L (0.51-1.02 mg/dL)
Albumin	19.2 g/L	1.92 g/dL	35-50 g/L (3.5-5.0 g/dL)
Calcium	4.48 mmol/L	17.92 mg/dL	2.20-2.80 mmol/L (8.8-11.2 mg/dL)
Ionized calcium	2.20 mmol/L	8.8 mg/dL	1.15-1.32 mmol/L (4.60-5.28)
Phosphate	0.98 mmol/L	3.03 mg/dL	0.80-1.5 mmol/L (2.48-4.64 mg/dL)
Vitamin D	49 nmol/L	19.6 ng/mL	50-100 nmol/L (20-40 ng/mL)
PTH	<9.4 ng/L	<9.4 pg/nL	9.4-66.0 ng/L (9.4-66.0 pg/nL)
PTH-rp	33 pmol/L		<0.6 pmol/L, < 3.4 pmol/L in pregnancy

Abnormal values are shown in bold font.

Abbreviations: PTH-rp: PTH-related peptide.

## Diagnostic Assessment

The hypercalcemia was accompanied by a suppressed PTH level, which raised the suspicion of PTH-rp-induced hypercalcemia, which was confirmed by the blood test. Imaging (magnetic resonance imaging abdomen and computed tomography thorax) showed no source for the production of PTH-rp except for the uterine myoma. Therefore, we hypothesized that the hypercalcemia was caused by the PTH-rp production in her giant myoma, which could be stimulated by the estrogen levels in pregnancy (6).

## Treatment

The patient was admitted to the intensive care unit. The first step in treating hypercalcemia during pregnancy is similar to the treatment of nonpregnant patients: hydration to achieve polyuria and thereby stimulate calciuresis. However, using loop diuretics is associated with reduced placental perfusion and is therefore relatively contraindicated in pregnancy (7). Calcitonin reduces serum calcium mainly by inhibiting osteoclast-mediated bone resorption and, to a lesser extent, by increasing renal calcium excretion. Calcitonin does not cross the placenta and is considered safe in pregnancy. Calcitonin can be administered subcutaneously or intramuscularly in a 4 to 8 IU/kg dosage every 6 hours. However, the effect of lowering calcium levels is transient and limited to 1.2 to 2.0 mg/dL (0.3-0.5 mmol/L) (3). Cinacalcet is a calcimimetic and binds to the calcium-sensing receptor, resulting in a decrease in PTH secretion. Therefore, cinacalcet is only effective in hypercalcemia due to hyperparathyroidism and thus not in our patient. In nonpregnant patients, bisphosphonates are the next step in the treatment of hypercalcemia. Bisphosphonates are not associated with congenital malformations. However, bisphosphonates cross the placenta and are associated with a mild decrease in gestational age, birth weight, and mild transient hypocalcemia and an increase in spontaneous abortions. Because bisphosphonates retain for years in the maternal and fetal bone tissue and long-term safety data is scarce, we refrained from administering bisphosphonates in our pregnant patient (1, 3, 8-11).

**Figure 2.**
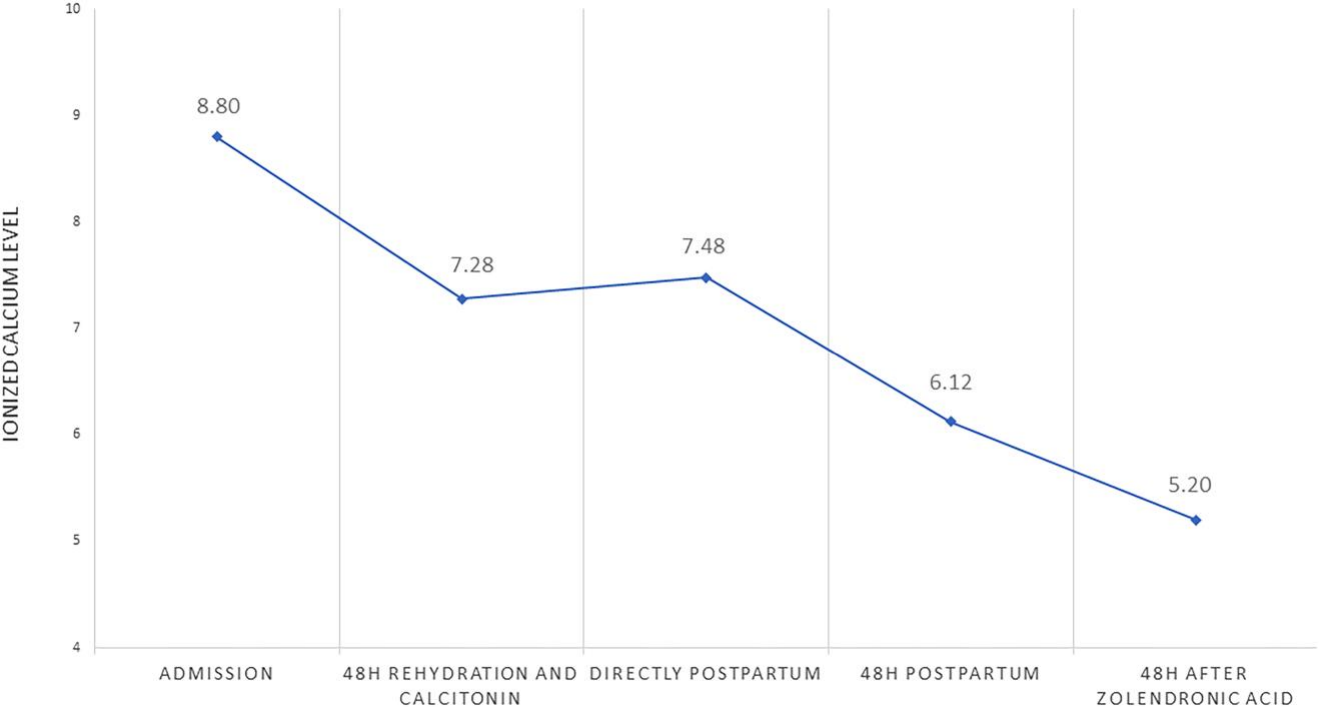
Course of ionized calcium level. Reference range: 4.60-5.28 mg/dL.

In our patient, hydration was started (6 L isotonic saline 0.9% per 24 hours) combined with calcitonin 400 IE (6 IE/kg) every 6 hours subcutaneously. After 48 hours of treatment, the ionized calcium levels had dropped to 7.28 mg/dL (1.82 mmol/L) (reference range 4.6-5.28 mg/dL 1.15-1.32 mmol/L), and her delirium improved (Fig. 2). The patient went into labor before the next therapeutic steps could be considered.

## Outcome and Follow-up

On day 3 of admission, at the gestational age of 31 weeks and 3 days, spontaneous labor started with the rupture of membranes and contractions. Despite hydration and calcitonin injections, at that time the ionized calcium level was 7.44 mg/dL (1.86 mmol/L) (reference range 4.6-5.28 mg/dL; 1.15-1.32 mmol/L). Because of the breech position of the fetus, an emergency cesarean section was performed, and a boy was born with a birth weight of 1920 grams, according to gestational age. Initially, the baby experienced severe difficulty in breathing due to airway obstruction because of his prematurity, for which short-term resuscitation was successfully performed. The ionized calcium level of the baby was 7.64 mg/dL (1.91 mmol/L) (reference range 3.80-6.00 mg/dL; 0.95-1.5 mmol/L) and normalized within 24 hours without any intervention. No hypocalcemia occurred, and his PTH levels were normal. Despite the difficult start of the baby due to prematurity, he was discharged after 5 weeks in good clinical condition.

**Figure 3.**
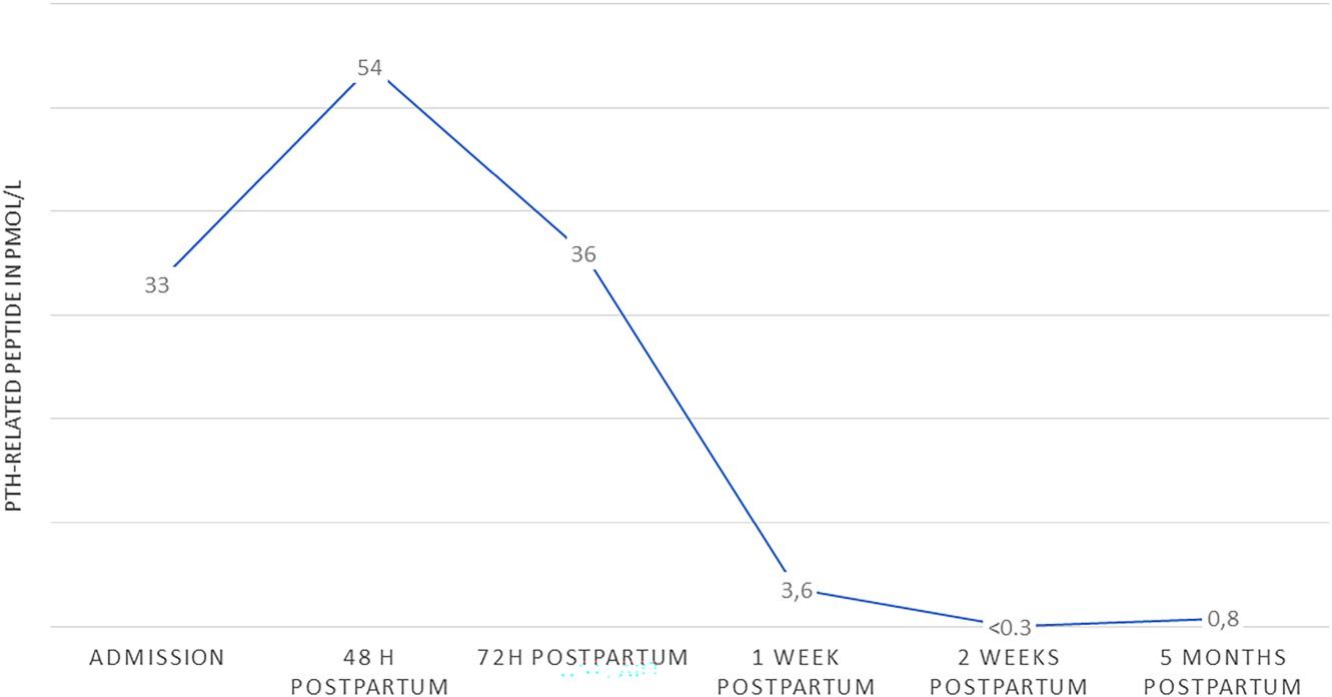
Course of PTH-related peptide (pmol/L). Reference range: <3.4 pmol/L in pregnancy, <0.6 pmol/L in non-pregnant.

Because the cesarean section was complicated by severe hemorrhage, the gynecologist decided to leave the myoma in situ. Postpartum, the ionized calcium level of the mother remained stable at 7.48 mg/dL (1.87 mmol/L) (reference range 4.6-5.28 mg/dL and 1.15-1.32 mmol/L). To induce forced diuresis, furosemide was started. After 2 days, the calcium was still 6.12 mg/dL (1.53 mmol/L), and 5 mg of zoledronic acid was added, after which calcium normalized (Fig. 2). Although we did not correct the vitamin D deficiency, the mother did not experience hungry bone syndrome. Because of her malnourished state, as well as the production of PTH-rp in lactating breast tissue, breastfeeding was discouraged. Postpartum PTH-rp levels initially peaked at 54 pmol/L (reference range: <0.6 pmol/L) on the second day and normalized 2 weeks postpartum. However, at 5 months postpartum, the PTH-rp was slightly increased (Fig. 3). We hypothesized that this could be due to estrogen stimulation of the myoma during the menstrual cycle. The calcium level remained within the reference range during follow-up. Thus far, the patient has chosen not to undergo a myomectomy.

## Discussion

We presented a rare case of PTH-rp production in a giant myoma, inducing hypercalcemia in pregnancy. We found 5 similar cases of uterine myomas that were humorally active during pregnancy causing hypercalcemia (12-15). In all 5 cases, the diagnosis was established on clinical grounds combined, with low PTH and high PTH-rp levels, the presence of a myoma and imaging ruling out other causes of PTH-rp production. A different treatment strategy was followed in every case. In the first case, hypercalcemia was found in the first trimester of pregnancy, and hydration, furosemide, calcitonin, and cinacalcet were administered without sufficient effect: the pregnancy was terminated at a gestational age of 15 weeks, and a myomectomy was performed. In the second case, hypercalcemia was also found in the first trimester; calcium levels could only be sufficiently lowered by hemodialysis. In the other 3 cases, the hypercalcemia was detected in the third trimester and was treated by hydration, loop diuretics, calcitonin, and in 2 cases bisphosphonates. In our case, we did not administer cinacalcet because we did not expect any calcium-lowering effects in the absence of hyperparathyroidism. We also refrained from administrating bisphosphonates and diuretics because of safety concerns (1, 3, 8-11). In our opinion, hydration and calcitonin are the cornerstones of treatment for PTH-rp-induced hypercalcemia.

Uterine myomas are common among women of reproductive age. Uterine myomas develop from uterine smooth muscle cells, which are regulated by estrogen, progesterone, and other growth factors. PTH-rp expression is increased in myoma tissue in comparison with normal myometrium. It is hypothesized that PTH-rp plays a role in regulating myoma growth and differentiation (5). Unfortunately, immunohistochemical staining of PTH-rp on the myoma, placenta, or amnion of our patient was not available. However, we could not find another source for the PTH-rp production on imaging. During pregnancy, mean PTH-rp rises from 0.81 in the first trimester to 2.01 pmol/L (reference range in pregnancy <3.4 pmol/L) at term and remains elevated in the first months postpartum, which is related to lactation (4, 16). The remarkable postpartum decline in PTH-rp level in our patient may be explained by the declining levels of estrogen and progesterone and, thus, the absence of stimulation of the myoma.

Despite the prompt treatment of hypercalcemia, we were not able to prevent preterm delivery. The risk of maternal and fetal complications is directly related to calcium concentration and, in particular, if the calcium concentration is >11.40 mg/dL (>2.85 mmol/L) (1-3). Maternal complications of hypercalcemia include miscarriage, preterm delivery, hyperemesis gravidarum, nephrolithiasis, pancreatitis, hypertension, preeclampsia, and renal insufficiency. Fetal complications of hypercalcemia during pregnancy include intrauterine growth restriction and death. Neonatal complications include symptomatic hypocalcemia and, rarely, permanent hypoparathyroidism due to longstanding exposure to hypercalcemia, leading to hypoplasia of the parathyroids. Because parathyroid disorders during pregnancy are rare, the prevalence of neonatal hypoparathyroidism is unknown (1-3). The lack of neonatal hypocalcemia in our case is remarkable because the mother was experiencing symptoms for a prolonged period of time (several weeks) and because of the degree of hypercalcemia. Because fetal development of the parathyroid glands takes place in the first 14 weeks of pregnancy, it could be hypothesized that not only the degree but also the timing of hypercalcemia in pregnancy is an important factor for the development of fetal hypoparathyroidism.

Fortunately, despite the difficult start, both mother and son are doing well.

## Learning Points

- Myomas can cause hypercalcemia during pregnancy due to PTH-rp production by estrogen stimulation.
- Diagnosis of hypercalcemia during pregnancy is challenging due to the rarity and nonspecific symptoms but vital because of the high maternal and neonatal morbidity and mortality in untreated hypercalcemia.
- Hydration and calcitonin injections are the cornerstones of the treatment of PTH-rp-induced hypercalcemia during pregnancy.

## Data Availability

Original data generated and analyzed for this case report are included in this published article.
